# The association between systemic immune-inflammation index and prostate-specific antigen: Results from NHANES 2003–2010

**DOI:** 10.1371/journal.pone.0313080

**Published:** 2024-11-21

**Authors:** Zhongqiu Tang, Shaojie Li, Mengjun Zeng, Lu Zeng, Zhaohui Tang

**Affiliations:** 1 Department of Oncology, The Central Hospital of Yongzhou, Yongzhou, Hu Nan, China; 2 Department of Neurosurgery, The Second Affiliated Hospital of Fujian Medical University, Quanzhou, Fujian, China; The University of the West Indies, JAMAICA

## Abstract

**Purpose:**

Current research has not extensively explored the correlation between Systemic Inflammatory Index (SII) and prostate-specific antibody (PSA) levels. This study aimed to investigate the relationship between the SII and PSA levels in American males aged > 40 years without prostate cancer.

**Methods:**

Data were obtained from the 2003–2010 National Health and Nutrition Examination Survey (NHANES). Patients without complete SII or PSA data were excluded. Multiple linear regression models were used to investigate the possibility of a linear association between the SII and PSA levels. Fitted smoothed curves and threshold effect analyses were used to characterize the nonlinear relationships.

**Results:**

The study included 5982 male participants over the age of 40 years from the United States. The average SII (mean ± standard deviation) was 562.78 ± 355.60. The mean value of PSA was 1.85 ± 3.24. The results showed that SII exhibited a positive correlation with PSA (β = 0.0005, 95% CI: (0.0002, 0.0007)), and an interaction test indicated that the effects of age, body mass index, hypertension, and diabetes were not significant for this positive correlation between SII and PSA (all P > 0.05). We discovered an inverted U-shaped connection between the SII and PSA with a turning point (K) of 1168.18 by using a two-segment linear regression model. To the left of the turning point, there was a positive connection between SII and PSA (β = 0.0009,95% CI: (0.0006, 0.0012); P < 0.0001).

**Conclusion:**

In the population of men over 40 years old without prostate cancer, SII and PSA exhibited a non-linear relationship. Specifically, there was a positive correlation between SII and PSA levels when the SII value was < 1168.18.

## Introduction

The 2023 U.S. Cancer Report suggests that the incidence of prostate cancer (PCa) declined by approximately 40% from 2007 to 2014, possibly due to a decline in the value of diagnosing localized tumors by testing for prostate-specific antigen (PSA) [1]. Numerous studies have demonstrated that variables other than PCa may also affect PSA concentrations, such as benign prostatic hyperplasia [2], demographics as well as lifestyle [3], antibiotics [4], and body mass index (BMI) [5]. These factors may lead to diagnostic bias.

Previous studies have reported that the triglyceride glucose index (TyG) and serum PSA concentrations are correlated in adult men in the United States. The PSA levels were lower in individuals with higher TyG indexes [6]. When blood albumin levels increased above 41 g, there was a negative correlation between serum albumin and PSA levels [7]. In a correlation study, Wei et al. used machine learning with XGBoost modeling to determine that among the selected variables, triglycerides were the most related to PSA [8]. Additionally, men with higher testosterone levels also have higher PSA levels [9]. However, there are no studies on the relationship between PSA levels and Systemic Immunoinflammatory Index (SII) in non-prostate cancer populations.

The SII is calculated based on platelets × neutrophils/lymphocytes and is a simple and robust index [10]. The relationship between inflammatory factors and prostate cancer remains controversial. One predictive indicator for men with advanced prostate cancer may be the neutrophil-to-lymphocyte count ratio (NLR) [11]. A meta-analysis suggested that higher pretreatment SII levels in patients with prostate cancer may be associated with lower overall survival (OS) and progression-free survival (PFS) [12]. However, preoperative inflammatory indicators in patients with prostate cancer were not correlated with worse prognosis in a prospective cohort [13]. The effects of other overlooked interactions, such as other characteristics including population, race, and age, could be the cause of this variation in the results. Thus, using information from the 2003 to 2010 National Health and Nutrition Examination Survey (NHANES) of American men 40 years of age and older without prostate problems, our goal was to examine the relationship between SII and PSA levels.

## Materials and methods

### Description of the survey

Cross-sectional data were obtained from the NHANES, a national study led by the National Center for Health Statistics (NCHS) and used to evaluate the nutritional and physical health status of the U.S populations [14]. An intricate multistage probabilistic strategy was employed to ensure that the sample taken was representative of the American population across the entire country. To obtain demographic, socioeconomic, and health-related data, the participants took part in household interviews. Physical and laboratory examinations were performed at the Mobile Examination Center (MEC). As these four NHANES cycles (two-year periods) were the only survey cycles with comprehensive PSA data, they were chosen to evaluate the relationship between SII and serum PSA levels. The four cycles spanned 2003–2010.

The NCHS Ethics Review Board authorized all NHANES study protocols, and each survey respondent signed an informed consent form. The public can access all comprehensive NHANES study designs and data from www.cdc.gov/nchs/nhanes/. The cross-sectional study reporting requirements and Strengthening the Reporting Observational Studies in Epidemiology (STROBE) protocols were adhered to.

### Research population

**Fig 1:**
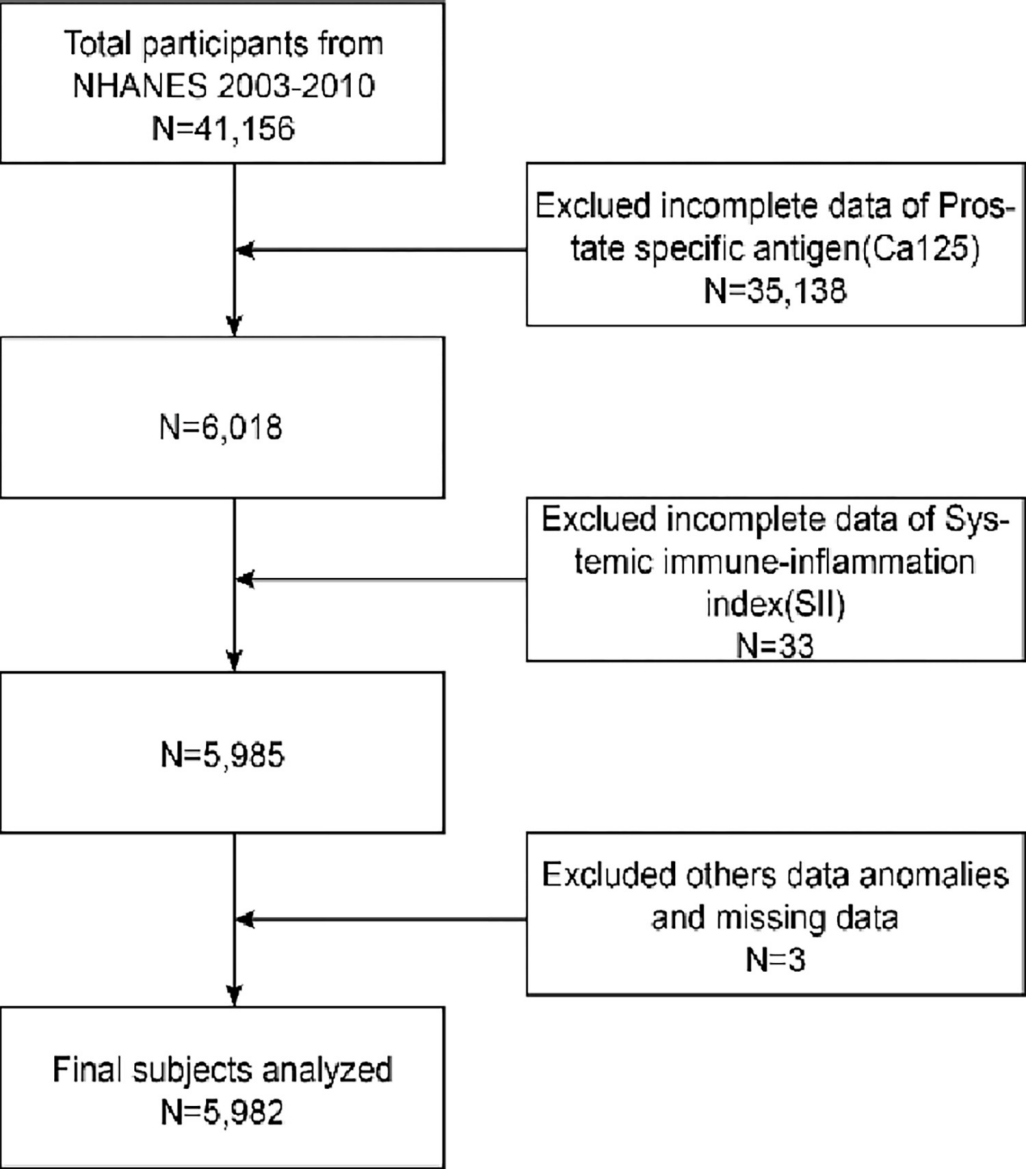
Flowchart of NHANES sample selection, 2003–2010.

Our exclusion criteria for the subjects we analyzed were (1) age < 40 years; (2) Missing complete data on PSA and SII; (3) Various factors affecting PSA (medications such as 5-ARIs, prostate enlargement, prostate inflammation, and infection, prostate biopsy within one week, urologic surgery within one month, history of prostate cancer, etc.). In this study, 41,156 individuals were initially recruited. After removing missing data on PSA (n = 35,138), SII (n = 33), and other anomalies (n = 3), we ultimately included 5,982 eligible participants aged > 40 years. (Fig 1).

### Definition of systemic immunoinflammatory index

**Fig 2:**
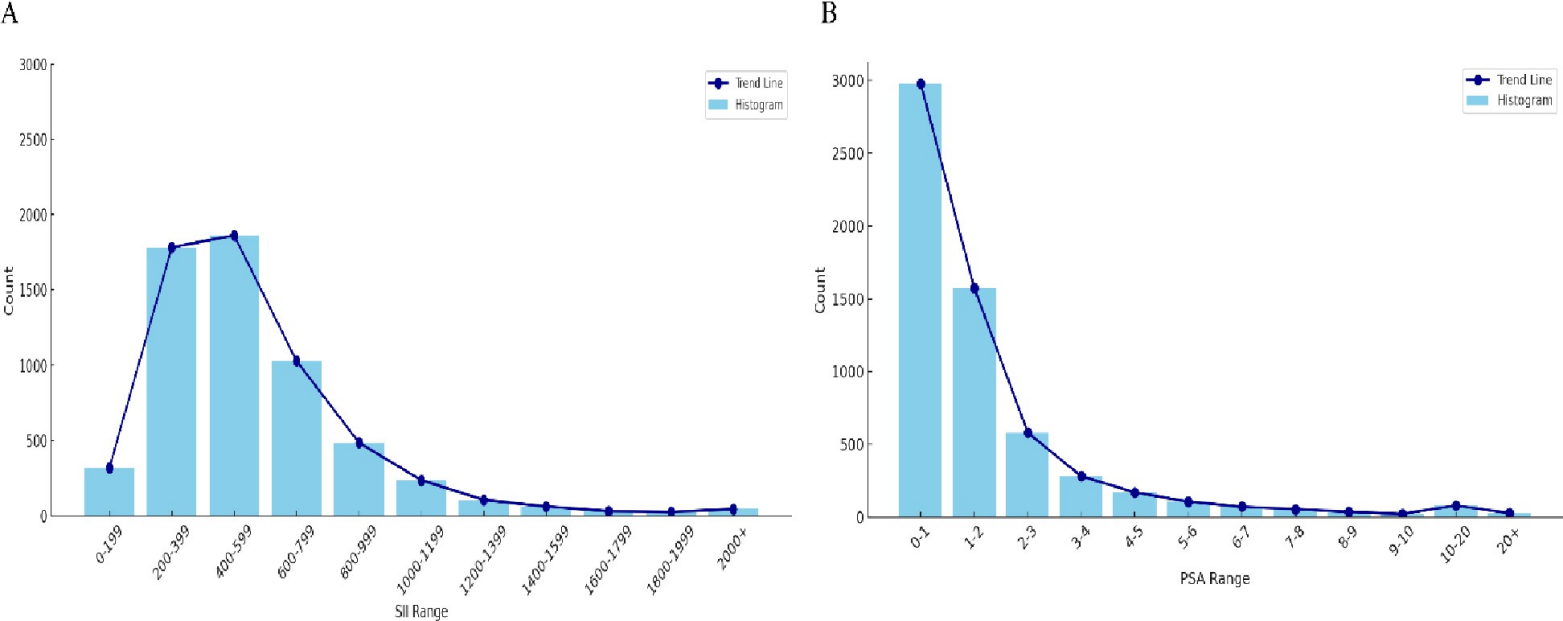
The respective distributions of the exposure and outcome variables. A) SII; B) PSA.

Blood samples were collected from participants at standardized mobile testing centers. Using an automated hematology analysis system (Coulter DxH 800 analyzer), the counts of lymphocytes, neutrophils, and platelets were determined using full blood counts and expressed in ×103 cells/μl. As reported in previous studies, we obtained the SII value by calculating the following formula: platelet count × neutrophil count/lymphocyte count [15,16]. The SII was selected as the exposure variable in our analysis. Fig 2A illustrates in detail the distribution of SII.

### Definition of PSA

The Beckman Access Immunoassay System’s Hybritech PSA technique, which automatically recognized the creation of light in reacted samples, was used to determine total PSA concentrations. The Access Hybritech test, which measures using a two-site immuno-enzymatic "sandwich" assay, was used to detect free PSA concentrations [17,18]. Serum total PSA levels were chosen as the outcome variable in our analysis. Fig 2B details the distribution of serum PSA.

### Covariates

Covariates that might have an impact on the relationship between the SII and PSA were also examined in our study; these included age, ethnicity, educational level, BMI, drinking status, urea nitrogen, cholesterol, lactate dehydrogenase, total bilirubin, triglycerides, serum uric acid, serum creatinine, history of hypertension, diabetes mellitus [7], coronary artery disease, angina pectoris, and tumor [19].

The above covariates can be broadly categorized into demographic data, laboratory indicators, and chronic disease history. Demographic data were collected through household interviews. BMI data were obtained from high-quality body measurements taken from the survey participants at the examination visit. BMI was categorized into BMI < 25,25 ≤ BMI < 30 and MBI ≥ 30 kg/m2, corresponding to normal weight, overweight, and obese populations among the participants, respectively [20]. Alcohol consumption was categorized as "no alcohol" and "at least 12 drinks a year" based on alcohol consumption in the year before the interview. Laboratory biochemical tests were analyzed using both the Beckman Sync LX20 and Beckman UniCel® DxC800 in parallel to obtain detailed biochemical indicators of the participants. Participants were interviewed face-to-face using a complex and detailed set of scales that were rigorously scrutinized and edited to ensure the accuracy of the data, resulting in a broad classification of chronic medical history as "yes" and "no." The Beckman Coulter® MAXM instrument was used perform a complete blood count on a blood specimen, obtaining the appropriate platelet (1,000 cells/μl), neutrophil (1,000 cells/μl), and lymphocyte (1,000 cells/μl) counts. All detailed measurement procedures for the above variables are publicly available at www.cdc.gov/nchs/nhanes/.

### Statistical analysis

The U.S. Centers for Disease Control and Prevention (CDC) recommendations were followed to conduct all statistical analyses. Appropriate NHANES sampling weights were used and a sophisticated multistage clustered survey design was considered in the analyses. In dealing with missing values, this study used the mean (median) to fill in continuous variables with a normal (skewed) distribution and plurality to fill in categorical variables. Continuous parameters were presented as means and standard deviations, and categorical variables were presented as percentages. Weighted Student’s t-tests (for continuous variables) or weighted chi-squared tests (for categorical variables) were used to evaluate differences between the SII (quartiles) groups. Three distinct models were examined using multiple regression equations to investigate the relationship between the SII and PSA levels. The covariates were not adjusted for in Model One. Model two takes race and age. Model three was adjusted for age, ethnicity, education level, BMI, blood urea nitrogen, cholesterol, lactate dehydrogenase, bilirubin, total cholesterol, triglycerides, blood urea nitrogen, creatinine, PSA, diabetes mellitus, hypertension, coronary heart disease, angina, tumor history, and drinking history. Subgroup analyses of the correlation between SII and PSA levels were performed. The stratification factors included age, BMI, hypertension, and diabetes. In addition, we included an interaction test to check for heterogeneity in the relationships between subgroups. The nonlinear connection between the SII and PSA levels was evaluated using a weighted generalized additive model (GAM) regression and smoothed curve fitting (penalized spline approach). Finally, a threshold effect analysis utilizing a two-stage linear regression model was used to further confirm the nonlinear association between the SII and PSA. Statistical significance was set at P < 0.05. For statistical analyses, we used R version 4.3.0 (http://www.R.project.org, R Foundation) and Empower software (www.empowerstats.com; X&Y Solutions, Inc., Boston, MA, USA).

## Results

### Baseline characteristics

The study comprised 5,982 individuals, with an average age(mean ± standard deviation) of 59.75 years (± 12.72). The interquartile ranges for SII were 11.88–342.73, 342.9–483.87, 483.88–682.62 and 682.88–5120 for quartiles 1, 2, 3, and 4, respectively. Participants’ total serum PSA (mean ± standard deviation) was 1.85 ± 3.24 and levels increased with increasing quartiles of SII (Q1: 1.59 ± 2.46; Q2: 1.74 ± 2.67; Q3: 1.78 ± 2.57; Q4: 2.28 ± 4.68, P < 0.0001). For each quartile, a higher interquartile range for SII corresponded to a higher interquartile range for total serum PSA levels. In addition, we found statistically significant differences in age, ethnicity, education level, BMI, drinking status, cholesterol, lactate dehydrogenase, total bilirubin, triglycerides, serum uric acid, serum creatinine, hypertension, angina, coronary artery disease, and cancer (all P < 0.05) among the different SII quartiles (Table 1). No statistically significant differences were observed between education level, serum urea nitrogen level, and history of diabetes (P > 0.05).

**Table 1:** Baseline characteristics of participants.

	Q1 (11.88–342.73)	Q2 (342.9–483.87)	Q3 (483.88–682.63)	Q4 (682.88–5120)	*P-*value
	n = 1496	n = 1495	n = 1495	n = 1496	
Age, years	58.00 (48.00–68.00)	58.00 (48.00–69.00)	59.00 (49.00–70.00)	61.00 (50.00–73.00)	<0.001
BMI(kg/m^2^)	28.09 (25.27–31.00)	28.38 (25.70–31.74)	28.39 (25.43–31.73)	28.05 (24.70–31.26)	0.003
Blood urea nitrogen (mmol/L)	13.00 (11.00–17.00)	14.00 (11.00–17.00)	14.00 (11.00–17.00)	14.00 (11.00–18.00)	<0.001
Cholesterol (mg/dL)	192.00 (166.00–222.00)	197.57 (171.00–226.50)	199.00 (170.00–227.00)	192.00 (166.00–222.00)	<0.001
Lactate dehydrogenase (U/L)	132.00 (116.00–150.00)	129.00 (116.00–146.00)	129.00 (116.00–144.00)	131.00 (117.00–147.00)	0.004
Bilirubin, total (umol/L)	0.80 (0.60–1.00)	0.80 (0.60–1.00)	0.80 (0.60–1.00)	0.80 (0.60–1.00)	0.013
Triglycerides (mg/dL)	128.00 (85.00–195.25)	146.00 (93.00–222.50)	140.00 (96.00–215.00)	129.00 (88.75–199.00)	<0.001
Uric acid (mg/dL)	5.90 (5.10–6.80)	6.00 (5.20–6.90)	6.00 (5.20–6.90)	6.00 (5.20–7.00)	0.135
Creatinine (mg/dL)	1.00 (0.90–1.13)	1.00 (0.88–1.11)	1.00 (0.90–1.10)	1.00 (0.90–1.18)	0.006
PSA (ng/mL)	0.90 (0.52–1.74)	0.98 (0.59–1.85)	1.00 (0.58–1.87)	1.10 (0.62–2.36)	<0.001
**Ethnicity, %**					<0.001
Mexican American	16.71%	19.53%	17.73%	15.04%	
Other Hispanic	7.82%	6.56%	6.29%	5.08%	
Non-Hispanic White	41.98%	51.71%	59.13%	63.97%	
Non-Hispanic Black	30.01%	18.33%	13.18%	11.56%	
Other Races	3.48%	58 (3.88%	3.68%	4.34%	
**Education level, %**					0.487
Less than junior high school	17.25%	18.19%	15.65%	16.18%	
Middle to high school	15.44%	12.64%	14.25%	15.17%	
High school graduate/GED or equivalent	22.19%	23.81%	24.48%	23.33%	
Some College or AA degree	23.73%	25.02%	24.21%	23.80%	
College Graduate or above	21.39%	20.33%	21.40%	21.52%	
**Diabetes mellitus, %**					0.194
No	81.35%	82.14%	82.41%	79.61%	
Yes	16.58%)	15.25%	14.85%	18.11%	
Borderline	2.07%)	2.61%	2.74%	2.27%	
**Hypertension, %**					<0.001
No	56.48%)	59.00%	55.25%	51.67%	
Yes	43.52%)	41.00%	44.75%	48.33%	
**Coronary heart disease, %**					0.007
No	91.18%)	92.58%	91.44%	89.04%	
Yes	8.82%)	7.42%	8.56%	10.96%	
**Angina, %**					0.017
No	94.92%)	96.32%	95.72%	93.98%	
Yes	5.08%)	3.68%	4.28%	6.02%	
**Tumor history, %**					<0.001
No	93.58%)	91.97%	90.70%	87.97%	
Yes	6.42%)	8.03%	9.30%	12.03%	
**Drinking history, %**					0.01
No	25.07%	23.61%	20.47%	24.87%	
At least 12 cups per year	74.93%	76.39%	79.53%	75.13%	

The population with normal weight, overweight, and obese individuals were classified as having a BMI of <25, 25–29.9, and ≥30 kg/m2, respectively.

### Higher SII may be associated with increased PSA

**Fig 3:**
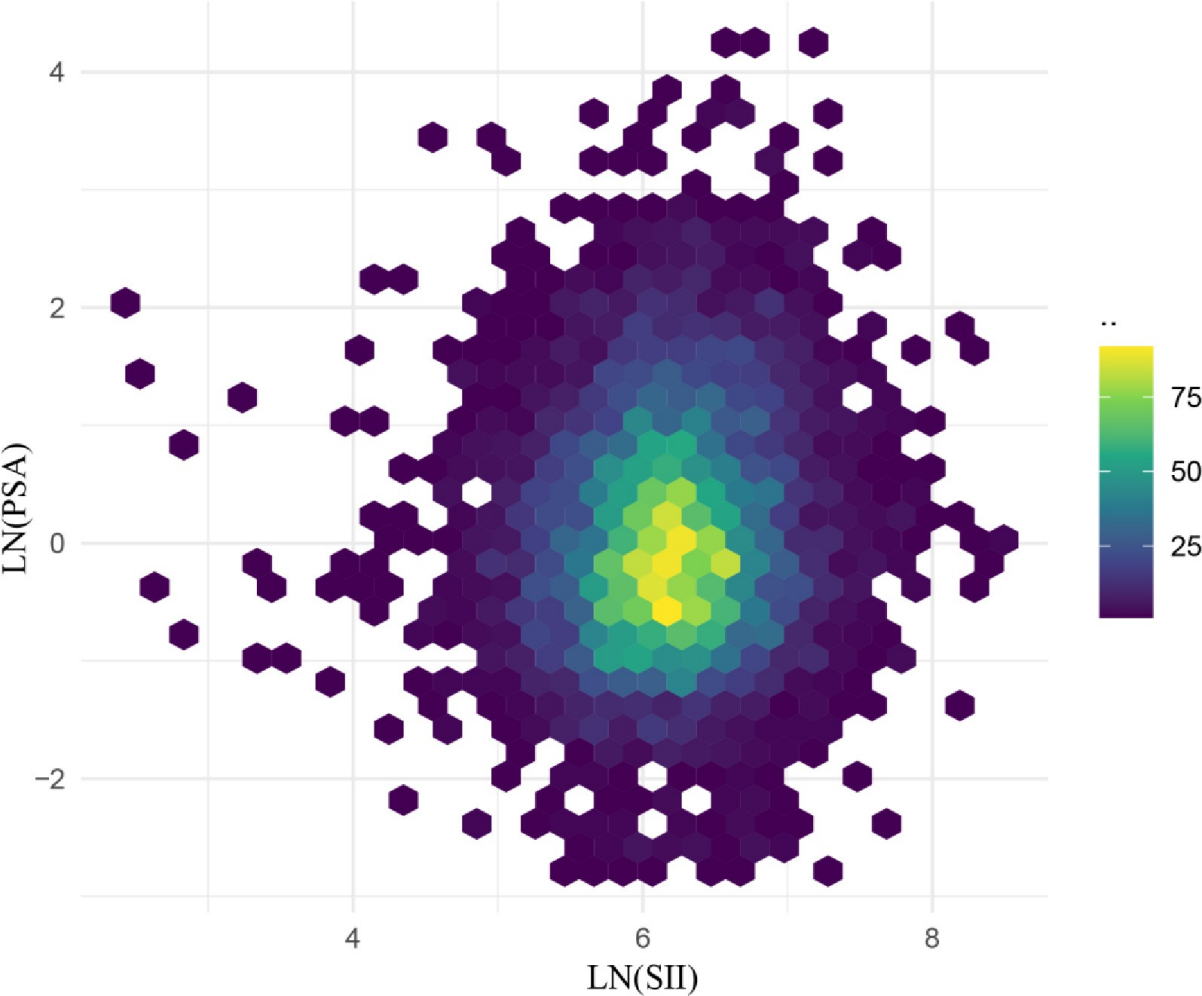
Distributional relationship between serum PSA and SII.

Table 2 shows the relationship between serum PSA levels and the SII. A strong positive association was exhibited between SII and serum total PSA in the crude (model one) and minimally adjusted (model two) models (model one:β = 0.0006, 95% CI: (0.0004, 0.0008); model two: β = 0.0005, 95% CI: (0.0002, 0.0007)). Fully adjusted for covariates (model three), PSA levels increased by 0.5 (ng/mL) for every 1,000 units of SII elevation. (Model three: β = 0.0005, 95% CI: (0.0002, 0.0007)). To investigate the correlation between SII and PSA more thoroughly, we converted SII into a categorical variable (quartiles) for analytical purposes. In the model with all adjustments, for each unit rise in SII compared with the lowest SII quartile (Q1), the PSA level in the highest SII quartile (Q4) rose by 0.6061 (ng/mL) (β = 0.6061, 95% CI: (0.3785, 0.8336)), P for trend < 0.000001). This indicated a stable, positive, and statistically significant correlation between increased SII and increased PSA levels (Table 2). In addition, Fig 3 demonstrates the distributional relationship between serum PSA and SII.

**Table 2:** Multiple regression equations for SII and PSA.

SII		β^d^ (95% CI^e^)	
	Crude model(Model one)^a^	Minimally adjusted model (Model two)^b^	Fully adjusted model (Model three)^c^
**Continuous**	0.0006 (0.0004, 0.0008)	0.0005 (0.0002, 0.0007)	0.0005 (0.0002, 0.0007)
**Categories**			
Q1	Reference	Reference	Reference
Q2	0.1515 (-0.0800, 0.3831)	0.2130 (-0.0111, 0.4370)	0.1968 (-0.0274, 0.4210)
Q3	0.1858 (-0.0458, 0.4174)	0.2297 (0.0040, 0.4554)	0.2065 (-0.0195, 0.4325)
Q4	0.6848 (0.4533, 0.9164)	0.6148 (0.3877, 0.8420)	0.6061 (0.3785, 0.8336)
*P* for trend	<0.000001	<0.000001	<0.000001

aModel one: There was no covariate adjustment.

bModel two: Adjusted for age and Ethnicity.

cModel three: Adjusted for Age、Ethnicity、Education level、BMI、Blood urea nitrogen、Cholesterol、Lactate dehydrogenase LDH、Bilirubin, total、Triglycerides、Blood urea nitrogen、Creatinine、PSA、Diabetes mellitus、Hypertension、Coronary heart disease、Angina、Tumor history、Drinking history.

dβ: Regression coefficient.

e95% CI:95% confidence interval.

### Nonlinear relationship between SII and total serum PSA

**Fig 4:**
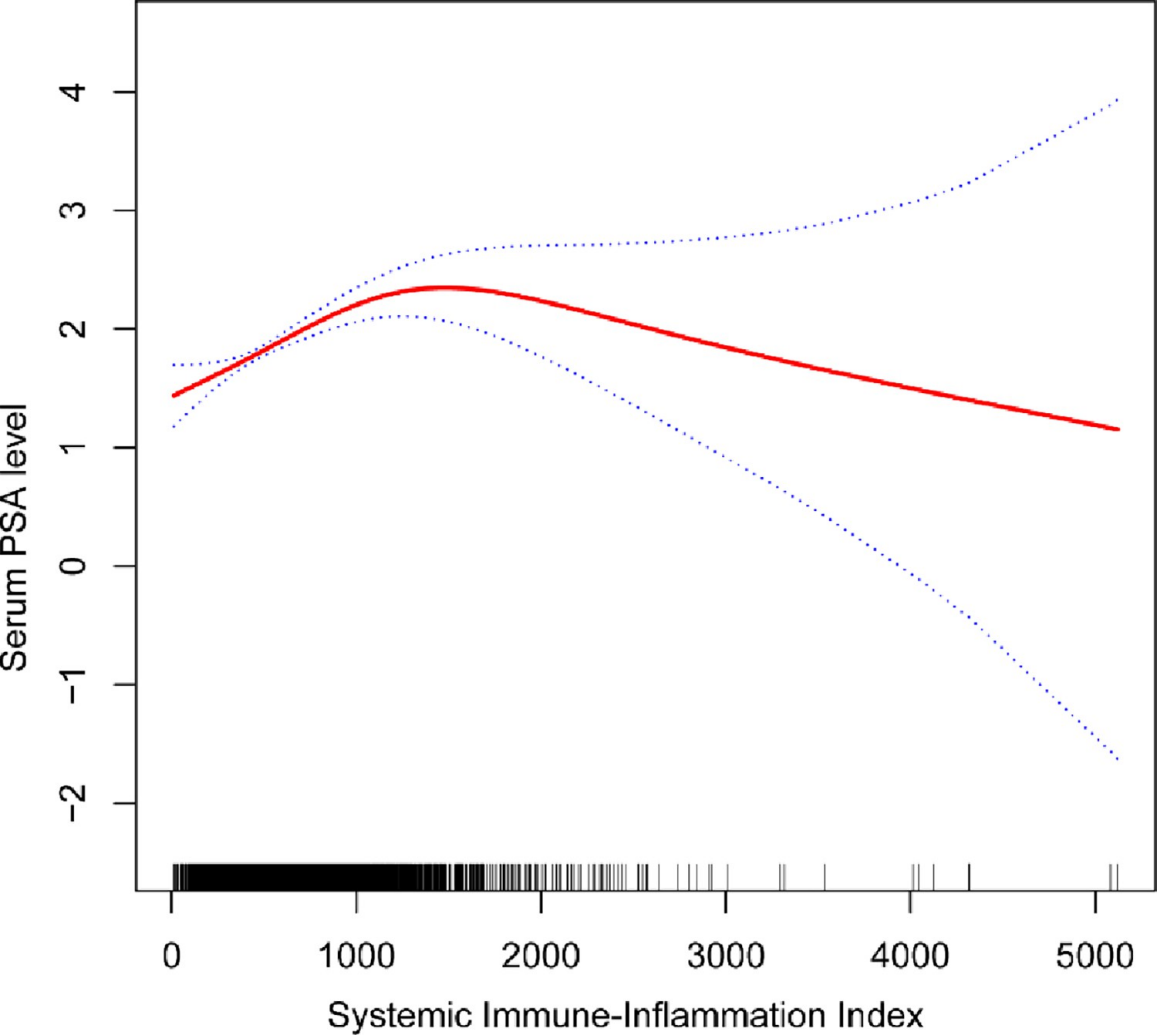
Correlation between SII and PSA. a: A sample is shown as a short line segment on the x-axis. b: The red line indicates the smooth curve fitted between the variables. The blue line shows the 95% confidence interval of the curve.

The unadjusted model’s multiple regression analysis results indicated a high positive correlation (β = 0.0006 95% CI: (0.0004, 0.0008)) between SII and PSA. This positive correlation was maintained after adjusting for covariates, as in the case of the partially adjusted model (β = 0.0005 95% CI (0.0002, 0.0007)) and the fully adjusted model (β = 0.0005 95% CI: (0.0002, 0.0007)). To further characterize the nonlinear connection between the SII and PSA, a smoothed curve was fitted (Fig 4).

The results indicated a nonlinear link between the SII and PSA by fitting a smooth curve to the association. This study further verified this nonlinear relationship using a two-segment linear regression model. An inverted U-shaped association with a turning point (K) of 1168.1818 was discovered between SII and PSA. SII and PSA showed a positive connection to the left of the turning point (β = 0.0009,95% CI: (0.0006, 0.0012); P < 0.0001). Nevertheless, at the right side of the inflection point, the connection between SII and PSA was not statistically significant (β = -0.0004,95% CI: (0.0009,0.0001); P = 0.1011). For the log-likelihood ratio test, the p-value was less than 0.001 (Table 3).

**Table 3:** Threshold effects analysis of PSA using a two-segment linear regression mode.

		Adjusted model^e^
Model one^a^	β**c** ^**(95% CI**^**d**^**),**^	0.0004 (0.0002, 0.0007)
	P ^**for trend**^	0.0001
Model two^b^	Breakpoint (K)	1168.1818
	β1 (< 1168.1818)	0.0009 (0.0006, 0.0012)
		<0.0001
	β2 (> 1168.1818)	-0.0004 (-0.0009, 0.0001)
		0.1011
	Difference in effect between 2 and 1	-0.0013 (-0.0020, -0.0006)
		0.0002
	Logarithmic likelihood ratio test *P* ^**value**^	<0.001

aModel one: Standard linear model.

bModel two: Two-piecewise linear model.

Cβ: regression coefficient.

d95% CI 95% confidence interval.

eAdjusted for Age、Ethnicity、Education level、BMI、Blood urea nitrogen、Cholesterol、Lactate dehydrogenase LDH、Bilirubin, total、Triglycerides、Blood urea nitrogen、Creatinine、PSA、Diabetes mellitus、Hypertension、Coronary heart disease、Angina、Tumor history、Drinking history.

### Subgroup analysis

**Fig 5:**
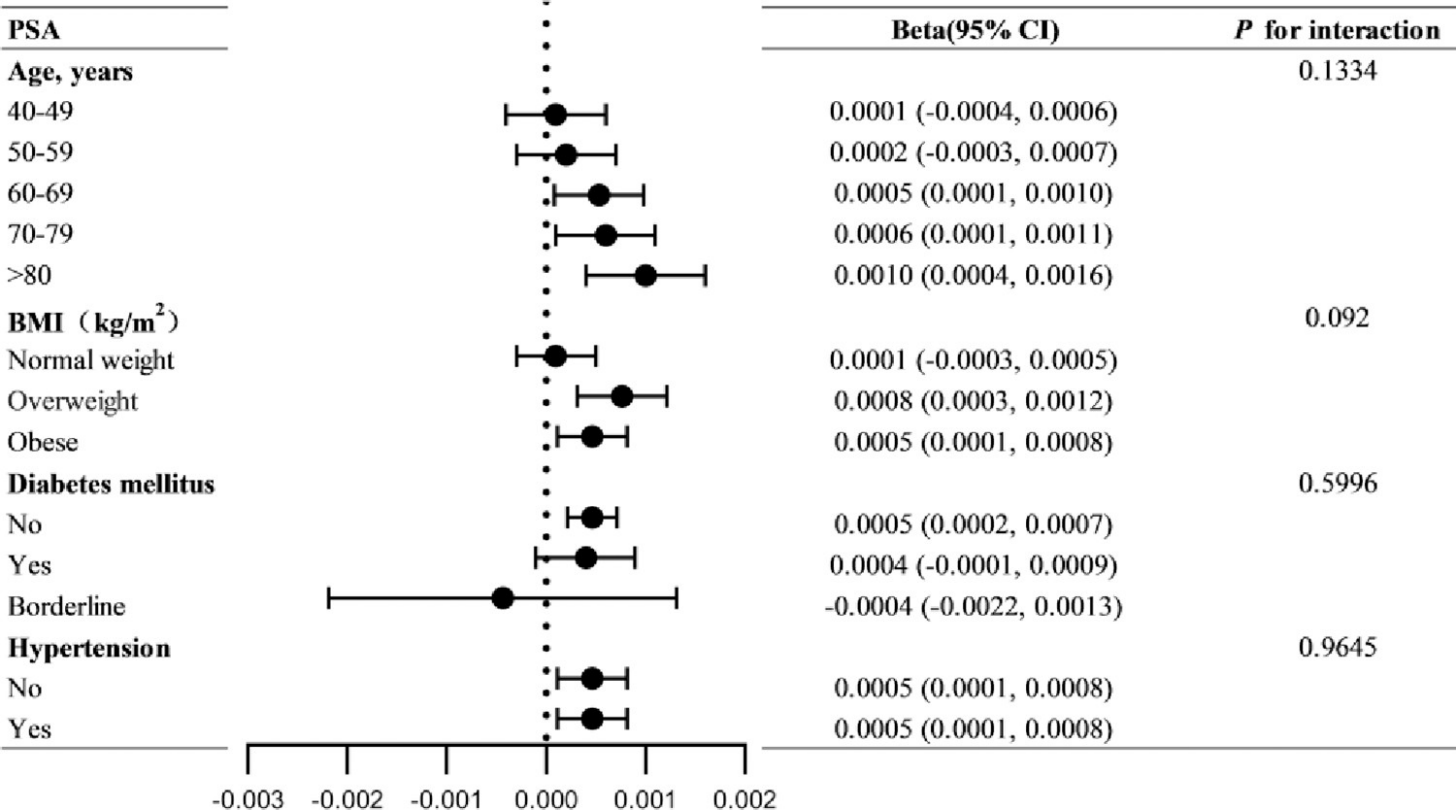
Subgroup analysis of the relationship between SII and PSA.

To determine whether the relationship between the SII and serum PSA levels remained constant across all populations, subgroup analyses and interaction tests stratified by hypertension, diabetes, age, and BMI were performed. Subgroup analyses demonstrated that, in the subgroups of hypertension, diabetes, age, and BMI, participants aged ≥ 60 years, overweight and obese, without diabetes, and with or without hypertension demonstrated statistical significance. Although not statistically significant, SII exhibited a negative correlation with PSA levels only in patients with prediabetes. In addition, the results of the interaction test revealed that the connection between the SII and PSA levels remained stable across populations. As shown in Fig 5, this positive association did not significantly interact with hypertension, diabetes, age, or BMI subgroups (all P>0.05). In summary, the association between the SII and PSA did not depend on populations with different hypertension, diabetes, age, or BMI. This positive correlation remained stable across a wide range of populations.

## Discussion

This study examined the association between SII and PSA levels between 2003 and 2010 in a sample of men aged > 40 years with non-prostate diseases in the US. At SII less than 1168.18, the results indicated a positive connection between SII and PSA levels.

It has long been established that inflammation and cancer are strongly related, with inflammation playing a major role in the emergence and spread of malignant tumors [21]. To better understand the inflammation-disease relationship, a novel composite inflammation indicator, SII, was developed. The SII was first defined in 2014 as the platelet count × neutrophil count/lymphocyte count. This composite parameter, which combines peripheral platelets, neutrophils, and lymphocytes, is a more comprehensive reflection of the inflammatory state of an organism than a single inflammatory indicator [15]. Since its inception, SII has been extensively examined in a variety of diseases. For example, SII is associated with tumor development and is an independent risk factor for CRC prognosis of colorectal cancer [22]. In addition, the SII predicts the prognosis of patients after radical resection of hepatocellular carcinoma and guides clinicians toward timely interventions in the form of columnar line graphs [10]. Among all systemic immune markers, SII was identified as an independent prognostic factor in patients with cervical cancer in a retrospective analysis. ROC curve analysis revealed that the SII had a larger area under the curve at 3 and 5 years than the neutrophil-lymphocyte ratio (NLR), monocyte-lymphocyte ratio (MLR), and platelet-lymphocyte ratio (PLR) [23]. A meta-analysis that included 7,087 patients noted that the SII may serve as a strong independent prognostic indicator in patients with postoperative bladder cancer patients [24]. In the diabetic subgroup, a cross-sectional investigation revealed that elevated SII levels were associated with a 137% greater risk of prostate cancer [21].

PSA testing has increased in popularity over the past 20 years. It helps with early prostate cancer detection, which lowers the risk of death in patients with prostate cancer [25–27]. Nevertheless, several studies have noted that various factors influence the PSA levels. Song et al. proposed that when dietary protein intake was more than 181.8 g, a positive correlation was observed between higher PSA levels and dietary protein intake. [28]. In a cross-sectional study that used data from the NHANES database, serum albumin and PSA levels were negatively correlated when blood albumin levels increased above 41 g/L [7]. Wang et al. found that chronic liver dysfunction reduces testosterone levels, leading to decreased PSA production [29].

The association between the SII and prostate cancer remains debatable. A meta-analysis suggested that a high SII in pretreatment prostate cancer patients may be associated with lower OS and PFS [12]. However, preoperative inflammatory indicators did not correlate with a worse prognosis in patients with prostate cancer in a prospective cohort [13]. Our cross-sectional study found that the SII was positively correlated with PSA at 1168.18. Subgroup analyses and interaction tests stratified by BMI, age, hypertension, and diabetes showed that the association between the SII and PSA levels remained stable in the population. Therefore, SII levels should be considered when interpreting PSA levels to avoid biased results.

Evidence suggests that up to one-quarter of all cancers are closely linked to chronic inflammatory diseases. However, the exact mechanism of this connection is still unknown [30]. Neutrophils and lymphocytes are innate immune cells that play a role in the body’s early reaction to tissue damage and contribute to the transformation and malignant development of cells [31]. This inflammation may arise before the development of malignant tumors or be induced by cancer, resulting in a pro-tumorigenic inflammatory microenvironment [32]. An inflammatory environment may stimulate cell proliferation [33]. Some T cell subtypes play a major role in adaptive immune responses that promote tumor growth [31]. Treg, Th17, and Th2 cells generate an immunosuppressive milieu and stimulate angiogenesis by generating a variety of cytokines. They are typically associated with tumor growth and poor prognosis [34,35]. It has been found that N2 neutrophils promote tumor angiogenesis, modify the extracellular matrix of the tumor microenvironment (TME) to promote tumor cell proliferation and control the early-stage biological characteristics of tumor cells, and promote tumor cell invasion and metastasis in the late-stage through the secretion of various inflammatory mediators (e.g., PGE2, CCL17, VEGF, Arg-1, iNOS, and B/MMP9 gelatinase) [36]. However, the specific mechanisms still need to be explored through extensive research.

This study, which used the NHANES database as its basis, attempted to improve the accuracy of the results by controlling for confounders and including a sizable and representative sample. However, this study had some limitations. First, even after several adjustments, we were unable to completely rule out the possibility that significant variables not included in the NHANES database would have impacted the results. Second, the exposure variable SII in this study was determined only by immune cell counts from a single blood test, which may not have revealed the subtle changes that may have occurred during the follow-up. Third, cross-sectional studies, by their nature, do not directly reflect causality and still require a significant number of prospective study tasks to ascertain.

## Conclusions

In summary, in a population of U.S. men aged > 40 years with non-prostate disorders, SII was nonlinearly related to PSA, with a positive correlation between SII and PSA levels when the SII value was less than 1168.18. The SII combined with PSA for the co-diagnosis of prostate disorders has the potential to become a more valuable assessment tool. However, further validation of our findings requires a larger multicenter prospective research cohort.

## References

[1] Maingi Shail, Dizon Don S. Cancer statistics, 2023[J]. CA: a cancer journal for clinicians, 2023, 73(1): 17–48. 36633525 10.3322/caac.21763

[2] Gudmundsson Julius, Jon K Sigurdsson Lilja Stefansdottir, Bjarni A Agnarsson, Helgi J Isaksson, Olafur A Stefansson et al. Genome-wide associations for benign prostatic hyperplasia reveal a genetic correlation with serum levels of PSA[J]. Nature Communications, 2018, 9(1): 4568. doi: 10.1038/s41467-018-06920-9 PMC6224563 30410027

[3] Kristal Alan R, Chi Chen, Tangen Catherine M, Goodman Phyllis J, Etzioni Ruth, Thompson Ian M. Associations of demographic and lifestyle characteristics with prostate-specific antigen (PSA) concentration and rate of PSA increase[J]. Cancer, 2006, 106(2): 320–328. 16342294 10.1002/cncr.21603

[4] Buddingh Karel T, Maatje Marlies G F, Putter Hein, Kropman René F, Pelger Rob C M. Do antibiotics decrease prostate-specific antigen levels and reduce the need for prostate biopsy in type IV prostatitis? A systematic literature review[J]. Canadian Urological Association Journal = Journal De l’Association Des Urologues Du Canada, 2018, 12(1): E25–E30. 10.5489/cuaj.4515 PMC5783704 29173276

[5] Zhao Ying, Zhang Yuting, Wang Xin, Lin Dandan, Chen Zongtao. Relationship between body mass index and concentrations of prostate specific antigen: a cross-sectional study[J]. Scandinavian Journal of Clinical and Laboratory Investigation, 2020, 80(2): 162–167. doi: 10.1080/00365513.2019.1703217 31855065

[6] Zhang Mengyu, Zhang Jiankang, Xing Zengshu. Association of TyG index with prostate-specific antigen (PSA) in American men: results from NHANES, 2003–2010[J]. Irish Journal of Medical Science, 2023. 10.1007/s11845-023-03431-5 PMC10808142 37340224

[7] Xu Kailiang, Yan Youji, Cheng Cong, Li Shiqin, Liao Yixiang 1, Jinmin Zeng et al. The relationship between serum albumin and prostate-specific antigen: A analysis of the National Health and Nutrition Examination Survey, 2003–2010[J]. Frontiers in Public Health, 2023, 11: 1078280. doi: 10.3389/fpubh.2023.1078280 36950094 PMC10025559

[8] Wei Chengcheng, Tian Liang, Jia Bo, Wang Miao, Xiong Ming, Hu Bo et al. Association between Serum Triglycerides and Prostate Specific Antigen (PSA) among U.S. Males: National Health and Nutrition Examination Survey (NHANES), 2003–2010[J]. Nutrients, 2022, 14(7): 1325. 35405939 10.3390/nu14071325 PMC9002993

[9] Peskoe Sarah B, Joshu Corinne E, Rohrmann Sabine, McGlynn Katherine A, Nyante Sarah J, Bradwin Gary et al. Circulating total testosterone and PSA concentrations in a nationally representative sample of men without a diagnosis of prostate cancer[J]. The Prostate, 2015, 75(11): 1167–1176. 25919471 10.1002/pros.22998 PMC4475411

[10] Hu Bo, Yang Xin-Rong, Xu Yang, Sun Yun-Fan, Sun Chao, Guo Wei et al. Systemic immune-inflammation index predicts prognosis of patients after curative resection for hepatocellular carcinoma[J]. Clinical Cancer Research: An Official Journal of the American Association for Cancer Research, 2014, 20(23): 6212–6222. 25271081 10.1158/1078-0432.CCR-14-0442

[11] Judith Stangl-Kremser Michael Sun, Ho Benedict, Thomas Joseph, Nauseef Jones T, Osborne Joseph R et al. Prognostic value of neutrophil-to-lymphocyte ratio in patients with metastatic castration-resistant prostate cancer receiving prostate-specific membrane antigen targeted radionuclide therapy[J]. The Prostate, 2023, 83(14): 1351–1357. 37424145 10.1002/pros.24597

[12] Meng Linghao, Yang Yujia, Hu Xu, Zhang Ruohan, Li Xiang. Prognostic value of the pretreatment systemic immune-inflammation index in patients with prostate cancer: a systematic review and meta-analysis[J]. Journal of Translational Medicine, 2023, 21(1): 79. 36739407 10.1186/s12967-023-03924-y PMC9898902

[13] Grogg Josias Bastian, Rizzi Gianluca, Gadient Jana, Wettstein Marian Severin, Affentranger Andres, Christian Daniel Fankhauser et al. Prognostic value of pretreatment inflammatory markers in localised prostate cancer before radical prostatectomy[J]. World Journal of Urology, 2023, 41(10): 2693–2698. 37749262 10.1007/s00345-023-04569-8 PMC10581955

[14] Curtin ester R, Mohadjer Leyla K, Dohrmann Sylvia M, Kruszon-Moran Deanna, Mirel Lisa B, Carroll Margaret D et al. National Health and Nutrition Examination Survey: sample design, 2007–2010[J]. Vital and Health Statistics. Series 2, Data Evaluation and Methods Research, 2013(160): 1–23. 25090039

[15] Qin Zheng, Li Hancong, Wang Liya, Geng Jiwen, Yang Qinbo, Su Baihai et al. Systemic Immune-Inflammation Index Is Associated With Increased Urinary Albumin Excretion: A Population-Based Study[J]. Frontiers in Immunology, 2022, 13: 863640. 35386695 10.3389/fimmu.2022.863640 PMC8977553

[16] Xie Ruijie, Liu Xiaozhu, Wu Haiyang, Liu Mingjiang, Zhang Ya. Associations between systemic immune-inflammation index and abdominal aortic calcification: Results of a nationwide survey[J]. Nutrition, metabolism, and cardiovascular diseases: NMCD, 2023, 33(7): 1437–1443. 37156667 10.1016/j.numecd.2023.04.015

[17] Liu Zhangcheng, Chen Chi, Yu Fuxun, Yuan Dongbo, Wang Wei, Jiao Ke et al. Association of Total Dietary Intake of Sugars with Prostate-Specific Antigen (PSA) Concentrations: Evidence from the National Health and Nutrition Examination Survey (NHANES), 2003–2010[J]. BioMed Research International, 2021, 2021: 4140767. 33506014 10.1155/2021/4140767 PMC7811566

[18] Miao ShuYing, Bao ChunXiang, Zhang YuanFeng, Wang LiJuan, Jin XiaoDong, Huang BiWu et al. Associations of the Geriatric Nutritional Risk Index with high risk for prostate cancer: A cross-sectional study[J]. Nutrition (Burbank, Los Angeles County, Calif.), 2023, 115: 112164. 37573791 10.1016/j.nut.2023.112164

[19] McDonald Alicia C, Vira Manish A, Vidal Adriana C, Gan Wenqi, Freedland Stephen J, Taioli Emanuela. Association Between Systemic Inflammatory Markers and Serum Prostate-Specific Antigen in Men without Prostatic Disease—The 2001–2008 National Health and Nutrition Examination Survey[J]. The Prostate, 2014, 74(5): 561–567. 10.1002/pros.22782 PMC4380881 24435840

[20] Culp Stephen, Porter Michael. The effect of obesity and lower serum prostate-specific antigen levels on prostate-cancer screening results in American men[J]. BJU international, 2009, 104(10): 1457–1461. 19522868 10.1111/j.1464-410X.2009.08646.x

[21] Hanahan Douglas, Weinberg Robert A. Hallmarks of cancer: the next generation[J]. Cell, 2011, 144(5): 646–674. 21376230 10.1016/j.cell.2011.02.013

[22] Chen Jian-Hui, Zhai Er-Tao, Yuan Yu-Jie, Wu Kai-Ming, Xu Jian-Bo, Peng Jian-Jun et al. Systemic immune-inflammation index for predicting prognosis of colorectal cancer[J]. World Journal of Gastroenterology, 2017, 23(34): 6261–6272. 28974892 10.3748/wjg.v23.i34.6261 PMC5603492

[23] Huang Huaping, Liu Qin, Zhu Lixia, Zhang Yan, Lu Xiaojuan, Wu Yawei et al. Prognostic Value of Preoperative Systemic Immune-Inflammation Index in Patients with Cervical Cancer[J]. Scientific Reports, 2019, 9(1): 3284. 30824727 10.1038/s41598-019-39150-0 PMC6397230

[24] Li Jinze, Cao Dehong, Huang Yin, Xiong Qiao, Tan Daqing, Liu Liangren et al. The Prognostic and Clinicopathological Significance of Systemic Immune-Inflammation Index in Bladder Cancer[J]. Frontiers in Immunology, 2022, 13: 865643. 35572533 10.3389/fimmu.2022.865643 PMC9097688

[25] Luo Zhumei, Wang Wei, Xiang Liyuan, Jin Tao. Association between the Systemic Immune-Inflammation Index and Prostate Cancer[J]. Nutrition and Cancer, 2023: 1–8. 10.1080/01635581.2023.2272800 37899742

[26] Neal David E, Donovan Jenny L, Martin Richard M, Hamdy Freddie C. Screening for prostate cancer remains controversial[J]. Lancet (London, England), 2009, 374(9700): 1482–1483. 19664817 10.1016/S0140-6736(09)61085-0

[27] Fritz H Schröder Jonas Hugosson, Carlsson Sigrid, Tammela Teuvo, Liisa Määttänen Anssi Auvinen et al. Screening for prostate cancer decreases the risk of developing metastatic disease: findings from the European Randomized Study of Screening for Prostate Cancer (ERSPC)[J]. European Urology, 2012, 62(5): 745–752. 22704366 10.1016/j.eururo.2012.05.068

[28] Song Jukun, Chen Chi, He Song, Chen Weiming, Su Jiaming, Yuan Dongbo et al. Is there a non-linear relationship between dietary protein intake and prostate-specific antigen: proof from the national health and nutrition examination survey (2003–2010) [J]. Lipids in Health and Disease, 2020, 19(1): 82. 10.1186/s12944-020-01234-6 PMC7195731 32359345

[29] Wang Anqi, Lazo Mariana, Carter H Ballentine, Groopman John D, Nelson William G, Platz Elizabeth A. Association between Liver Fibrosis and Serum PSA among U.S. Men: National Health and Nutrition Examination Survey (NHANES), 2001–2010[J]. Cancer Epidemiology, Biomarkers & Prevention: A Publication of the American Association for Cancer Research, Cosponsored by the American Society of Preventive Oncology, 2019, 28(8): 1331–1338. 10.1158/1055-9965.EPI-19-0145 PMC6677604 31160348

[30] Murata Mariko. Inflammation and cancer[J]. Environmental Health and Preventive Medicine, 2018, 23(1): 50. 30340457 10.1186/s12199-018-0740-1 PMC6195709

[31] Zhao Huakan, Wu Lei, Yan Guifang, Chen Yu, Zhou Mingyue, Wu Yongzhong et al. Inflammation and tumor progression: signaling pathways and targeted intervention[J]. Signal Transduction and Targeted Therapy, 2021, 6: 263. 34248142 10.1038/s41392-021-00658-5 PMC8273155

[32] Guthrie Graeme J K, Charles Kellie A, Roxburgh Campbell S D, Horgan Paul G, McMillan Donald C, Clarke Stephen J. The systemic inflammation-based neutrophil-lymphocyte ratio: experience in patients with cancer[J]. Critical Reviews in Oncology/Hematology, 2013, 88(1): 218–230. 23602134 10.1016/j.critrevonc.2013.03.010

[33] Cai Tommaso, Santi Raffaella, Tamanini Irene, Galli Ilaria Camilla, Perletti Gianpaolo, Johansen Truls E Bjerklund et al. Current Knowledge of the Potential Links between Inflammation and Prostate Cancer[J]. International Journal of Molecular Sciences, 2019, 20(15): 3833. 31390729 10.3390/ijms20153833 PMC6696519

[34] Shrihari T G. Innate and adaptive immune cells in Tumor microenvironment[J]. The Gulf Journal of Oncology, 2021, 1(35): 77–81. 33716216

[35] Fleming Christopher, Morrissey Samantha, Cai Yihua, Yan Jun. γδ T Cells: Unexpected Regulators of Cancer Development and Progression[J]. Trends in Cancer, 2017, 3(8): 561–570. 28780933 10.1016/j.trecan.2017.06.003 PMC5551453

[36] Ocana Alberto, Nieto-Jiménez Cristina, Pandiella Atanasio, Templeton Arnoud J. Neutrophils in cancer: prognostic role and therapeutic strategies[J]. Molecular Cancer, 2017, 16(1): 137. 28810877 10.1186/s12943-017-0707-7 PMC5558711

